# Evaluation of the prognostic value of lymphadenectomy for low-grade serous ovarian cancer: A case-control multicenter retrospective study

**DOI:** 10.1016/j.tranon.2022.101476

**Published:** 2022-07-04

**Authors:** Zhongshao Chen, Ran Chu, Yuanming Shen, Qin Yao, Jingying Chen, Tianyu Qin, Li Li, Gang Chen, Qinglei Gao, Chaoyang Sun, Li Song, Junting Li, Penglin Liu, Xiyu Pan, Jingnan Li, Xiaoying Zhu, Li Zhang, Xu Qiao, Ding Ma, Beihua Kong, Kun Song

**Affiliations:** a Department of Obstetrics and Gynecology, Qilu Hospital, Cheeloo College of Medicine, Shandong University, Jinan, Shandong, PR China; b Department of Gynecology, Women's Hospital School, Medicine Zhejiang University, Hangzhou, Zhejiang, PR China; c Department of Gynecology, the Affiliated Hospital of Qingdao University, Qingdao, Shandong, PR China; d Department of Obstetrics and Gynecology, Tongji Hospital, Tongji Medical College, Huazhong University of Science and Technology, Wuhan, Hubei, PR China; e School of Control Science and Engineering, Shandong University, Jinan, Shandong, PR China; f Department of Obstetrics and Gynecology, Qilu Hospital of Shandong University, Jinan, Shandong, PR China; g Gynecology oncology key laboratory, Qilu Hospital of Shandong University, Jinan, Shandong, PR China

**Keywords:** Low-grade serous ovarian cancer, Lymphadenectomy, Disease-free survival, Overall survival

## Abstract

**Background:**

The prognostic value of lymphadenectomy in low-grade serous ovarian cancer (LGSOC) remains uncertain.

**Materials and methods:**

A retrospective analysis of 155 patients with LGSOC who underwent surgery over a ten-year period (2011–2020) was performed. The propensity score matching (PSM) algorithm was performed between the lymphadenectomy and no lymphadenectomy groups, and Kaplan-Meier analyses were conducted to evaluate clinical prognosis. Finally, univariate and multivariate Cox proportional hazards regression analyses were performed to analyze high-risk factors associated with clinical prognosis.

**Results:**

In the pre-PSM cohort, 110 (71.0%) patients underwent lymphadenectomy. Of these, 54 (34.8%) experienced recurrence, and 27 (17.4%) died. There were statistical differences in disease-free survival (DFS) (*P* = 0.018) and overall survival (OS) (*P* = 0.016) in the post-PSM cohort. In the subgroup analysis, there were no statistically significant differences in DFS (*P* = 0.449) or OS (*P* = 0.167) in the FIGO I/II cohort. However, in the FIGO III/IV cohort, DFS (*P* = 0.011) and OS (*P* = 0.046) were statistically different between the two groups. Age > 50 years, FIGO stage III/IV, and suboptimal cytoreductive surgery were risk factors associated with prognosis. In the lymphadenectomy group, the histological status of pelvic lymph nodes had no significant effect on DFS (*P* = 0.205) or OS (*P* = 0.114).

**Conclusion:**

Lymphadenectomy was associated with DFS and OS, particularly in patients with advanced LGSOC patients. Age > 50 years, advanced FIGO stage III/IV, and suboptimal cytoreductive surgery were high-risk factors associated with clinical prognosis in patients with LGSOC.

## Highlights

• The prognostic value of lymphadenectomy in LGSOC is still uncertain.
• A multicenter retrospective analysis of 155 patients with LGSOC was performed.
• Lymphadenectomy was associated with DFS and OS, especially in advanced LGSOC.
• Age, the FIGO stage and suboptimal cytoreductive surgery are high-risk factors.

## Introduction

Ovarian cancer is the eighth most common malignancy and the eighth deadliest malignancy in women. According to the 2020 Global Cancer Data Report, there are 313,959 new cases of ovarian cancer (3.4%) and 207,252 deaths (4.7%) annually [1]. Histologically, epithelial ovarian cancers account for 90% of all ovarian cancers, with serous ovarian cancer being the most common type. Serous ovarian cancer is divided into high-grade serous ovarian cancer (HGSOC) and low-grade serous ovarian cancer (LGSOC), according to the two-tier grading system [2], [3], [4], [5], [6]. LGSOC accounts for approximately 6–10% of epithelial ovarian cancers [4,5,[7], [8], [9]]. Compared to HGSOC, LGSOC is diagnosed at a younger age and has a better prognosis; however, it is relatively chemoresistant [4,[10], [11], [12], [13], [14]].

Since LGSOC is a rare malignant ovarian tumor, clinical guidance for patients with LGSOC is mainly based on retrospective studies and subgroup analyses of ovarian cancer clinical trials [4,15,16]. During clinical treatment, the surgical management of ovarian cancer requires a hysterectomy, bilateral salpingo-oophorectomy, omentectomy, and visible resection of metastatic lesions [14,17,18]. Additionally, primary maximal cytoreductive surgery is of paramount importance for the clinical prognosis of patients with advanced LGSOC [7,17]. Previous studies have reported that approximately 20–70% of patients with ovarian cancer have lymph node metastasis, and this proportion gradually increases with the International Federation of Gynecology and Obstetrics (FIGO) stage [17,[19], [20], [21]]. However, whether lymphadenectomy should be performed during surgery remains inconclusive. A large randomized trial from 2019 (the LION study) reported that for patients with advanced ovarian cancer, systematic pelvic and para-aortic lymphadenectomy had no effect on survival and increased the risk of developing postoperative complications [22]. However, most patients in the LION study had HGSOC. There is still no conclusive clinical evidence regarding the clinical benefits of lymphadenectomy in patients with LGSOC [23,24].

The aim of the our study was to conduct propensity score matching (PSM) analyses to further evaluate the prognostic value of lymphadenectomy in patients with LGSOC at different FIGO stages. Our results provide a more individualized reference for surgical decision-making during clinical precision treatment.

## Patients and methods

### Study population

We retrospectively reviewed 199 patients with LGSOC who underwent surgery between 2011 and 2020 at the following four medical centers: Qilu Hospital of Shandong University, the Affiliated Hospital of Qingdao University, the Women's Hospital School of Medicine at Zhejiang University, and the Tongji Hospital at the Tongji Medical College of the Huazhong University of Science and Technology. Among them, 155 patients had complete follow-up data, whereas 44 patients had missing information or were lost to follow-up. All patients had a clear pathological diagnosis of LGSOC and underwent surgery for the initial treatment. Patients who did not undergo surgery, had no pathological results, or had other systemic malignancies were excluded.

### Data collection

Clinical characteristics, such as age at diagnosis, tumor size, preoperative serum carbohydrate antigen 125 (CA-125) level (U/mL), FIGO stage (2014) [25], surgical method and range, intraoperative pathology, presence of ascites, postoperative routine pathology results, postoperative pathological stage and adjuvant therapy, duration of follow-up, and survival outcomes were included in the analysis. The size of the largest residual tumor and postoperative pathological stage were evaluated according to surgical records and related pathological results. Patients with a maximum residual tumor diameter <1 cm were considered to have undergone optimal cytoreductive surgery, whereas patients with a maximum residual tumor diameter ≥1 cm were considered to have undergone suboptimal cytoreductive surgery.

### Outcomes

The primary outcomes evaluated were disease-free survival (DFS) and overall survival (OS). DFS was defined as the time from surgery to the first occurrence of disease progression, recurrence, or death due to the disease. If none of these events occurred, the date of last follow-up was used. OS was calculated as the time from surgery to death or the last follow-up if the patient was still alive. Recurrence was defined as elevated CA-125 levels and/or imaging abnormalities on transvaginal ultrasound, computed tomography (CT), and/or positron emission tomography (PET).

### Statistical analyses

**Supplementary Fig. S1** shows a flowchart of the statistical analyses. Patients were divided into the lymphadenectomy group and the no lymphadenectomy group according to whether pelvic and/or para-aortic lymphadenectomy was performed during the operation. The chi-square test was used to compare the clinical characteristics between the two groups. To ensure that the clinical characteristics were scientifically balanced between the two groups and to better evaluate the impact of lymphadenectomy on the patients’ clinical outcomes, we adopted the PSM algorithm. The characteristics with a *P* value < 0.20 after the chi-square test were matched using PSM, and 0.02 was set as the match tolerance value. These propensity scores were used to match the patients in the two groups at a fixed ratio of 1:1.

Kaplan-Meier analyses were conducted to evaluate the effects of lymphadenectomy on DFS and OS in the pre- and post-PSM cohorts. Univariate Cox proportional hazards regression analysis was used to screen for high-risk factors associated with DFS and OS. Characteristics with a *P* value < 0.15 were included in the multivariate Cox proportional hazards regression analysis. The results are represented using hazard ratios (HRs), 95% confidence intervals (CIs), and *P* values.

To further evaluate the effect of lymphadenectomy on DFS and OS in patients with LGSOC at different FIGO stages, we conducted a subgroup analysis. Patients were divided into FIGO stage I and II disease groups and FIGO stage III and IV disease groups, and PSM analyses were performed as previously described. Kaplan-Meier analyses were conducted to explore the effect of lymphadenectomy on DFS and OS according to the FIGO stage.

All statistical analyses were conducted using SPSS (IBM SPSS Statistics, V.25.0, IBM Corporation, Armonk, New York, USA). Statistical significance was set at *P* < 0.05.

## Results

### Patients’ clinical characteristics

This study included 155 patients with LGSOC from four medical centers. The median age was 47 years (range, 21–79 years), and 98 patients (63.2%) were premenopausal. There were 52 patients (33.5%) with stage I disease, 12 (7.7%) with stage II disease, 83 (53.5%) with stage III disease, and 8 (5.2%) with stage IV disease. Most of the 64 patients in the early stage underwent staged surgery, including hysterectomy, bilateral salpingo-oophorectomy, and omentectomy. Among the 91 advanced patients, most of the other patients underwent cytoreductive surgery, including hysterectomy, bilateral salpingo-oophorectomy, omentectomy, and resection of visible macroscopic lesions. Fifty-four patients (59.3%) were considered to have received optimal cytoreductive surgery (maximum diameter of the residual lesion < 1 cm).

Among the 110 patients (71.0%) who underwent pelvic and/or para-aortic lymph node dissection (LND) 30 (27.3%) had pathological evidence of lymph node metastasis. Thirty-seven of these 110 patients underwent pelvic LND (33.6%), and 72 underwent pelvic LND with para-aortic LND (65.5%). One 21-year-old patient underwent only para-aortic LND and had pathological findings of carcinoma. Among the 30 patients with lymph node metastasis, 11 (36.7%) had pelvic lymph node metastasis and para-aortic lymph node metastasis, 13 (43.3%) had pelvic lymph node metastasis, and six (20.0%) had para-aortic lymph node metastasis. Supplementary **Table S1** lists the related results.

Platinum-based adjuvant chemotherapy was administered to 134 (86.5%) patients. Fifty-four patients (34.8%) experienced disease progression or recurrence, of whom 45 (83.3%) had FIGO stage III or IV disease. The median DFS was 65 months. Twenty-seven patients (17.4%) died postoperatively because of the disease or other complications, 22 (81.5%) of whom had FIGO stage III or IV disease. The median OS was 90 months.

### Propensity score matching analysis

Table 1 shows the characteristics of patients in the pre- and post-PSM cohorts. In the pre-PSM cohort, statistically significant differences were observed in age (*P* = 0.024), FIGO stage (*P* = 0.018), CA-125 level (*P* = 0.014), cytoreductive surgery (*P* = 0.041), and adjuvant therapy (*P* = 0.044). PSM of the two cohorts was conducted at a ratio of 1:1. A total of 78 women were included in the post-PSM cohort: 39 (50.0%) had undergone lymphadenectomy and 39 (50.0%) had not. In the post-PSM cohort, the basic patient characteristics were not significantly different (*P* > 0.05).

**Table 1:** Characteristics of patients in the pre- and post-PSM cohorts.

	Before Matching (*n* = 155)			After Matching (*n* = 78)		
Characteristic	Lymphadenectomy (*n* = 110)	No lymphadenectomy (*n* = 45)	*P* value	Lymphadenectomy (*n* = 39)	No lymphadenectomy (*n* = 39)	*P* value
**Age, year**			0.024			0.496
≤50	75 (68.2)	22 (48.9)		19 (48.7)	22 (56.4)	
>50	35 (31.8)	23 (51.1)		20 (51.3)	17 (43.6)	
**FIGO (2014)**			0.018			0.804
I and II	52 (47.3)	12 (26.7)		11 (28.2)	12 (30.8)	
III and IV	58 (52.7)	33 (73.3)		28 (71.8)	27 (69.2)	
**CA-125, U/mL**			0.014			1.000
≤35	26 (23.6)	3 (6.7)		4 (10.3)	3 (7.7)	
>35	84 (76.4)	42 (93.3)		35 (89.7)	36 (92.3)	
**Operation method**			0.272			0.745
Laparotomy	87 (79.1)	39 (86.7)		34 (87.2)	33 (84.6)	
Laparoscopy	23 (20.9)	6 (13.3)		5 (12.8)	6 (15.4)	
**Tumor size, cm**			0.855			0.068
≤8	58 (52.7)	23 (51.1)		13 (33.3)	22 (56.4)	
>8	52 (47.3)	22 (48.9)		26 (66.7)	17 (43.6)	
**Pathological consistency**			0.691			0.659
Consistent	72 (65.5)	27 (60.0)		25 (64.1)	23 (59.0)	
Not consistent	22 (20.0)	9 (20.0)		9 (23.1)	8 (20.5)	
Unknown	16 (14.5)	9 (20.0)		5 (12.8)	8 (20.5)	
**Debulking surgery**			0.041			0.808
Optimal (<1 cm)	86 (78.2)	28 (62.2)		27 (69.2)	26 (66.7)	
Suboptimal (≥1 cm)	24 (21.8)	17 (37.8)		12 (30.8)	13 (33.3)	
**Ascites cytology**			0.204			0.536
Positive	16 (14.5)	11 (24.4)		4 (10.3)	7 (23.1)	
Negative	37 (33.6)	10 (22.2)		12 (30.8)	9 (17.9)	
Unknown	57 (51.8)	24 (53.3)		23 (59.0)	23 (59.0)	
**Adjuvant therapy**			0.044			0.784
None	11 (10.0)	10 (22.2)		8 (29.5)	9 (23.1)	
Chemotherapy	99 (90.0)	35 (77.8)		31 (70.5)	30 (76.9)	

Values are presented as n (%).

PSM, propensity score matching; FIGO, International Federation of Gynecology and Obstetrics; CA-125, carbohydrate antigen 125.

### Kaplan-Meier analyses of DFS and OS

**Fig. 1:**
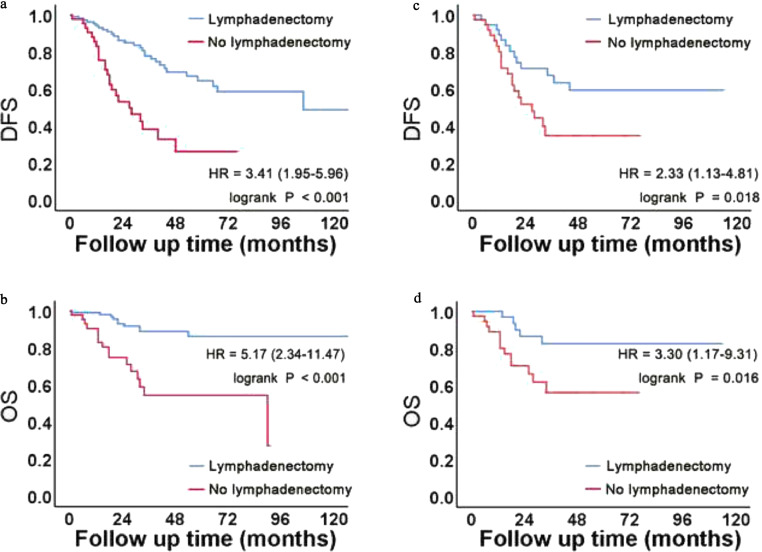
Kaplan-Meier curves for the pre- and post-PSM cohorts. (A, B) In the pre-PSM cohort, Kaplan-Meier survival analysis revealed that lymphadenectomy had a protective effect on DFS and OS. (C, D) After clinical characteristic matching, we found statistical differences in DFS and OS between the two groups.

Kaplan-Meier survival curves for OS and DFS according to lymphadenectomy status are presented in Fig. 1. In the pre-PSM cohort, Kaplan-Meier analyses showed that lymphadenectomy had a significant protective effect on DFS (*P* < 0.001) and OS (*P* < 0.001). The median DFS of the two pre-PSM cohorts was 106 and 27 months, respectively, and the median OS of the no lymphadenectomy group was 90 months. The results are shown in Figs. 1**A** and 1**B**. In the post-PSM cohort, there were statistically significant differences between the lymphadenectomy and no lymphadenectomy groups for DFS (*P* = 0.018) and OS (*P* = 0.016). The median DFS in the no lymphadenectomy group was 27 months. The results are shown in Fig. 1**C** and **D**.

### Univariate and multivariate Cox proportional hazards analyses for DFS and OS

Univariate and multivariate Cox proportional hazards regression analyses were performed for the post-PSM cohort. Univariate Cox proportional hazards regression analysis showed that age ≤ 50 years (*P* = 0.020), FIGO stage I or II (*P* = 0.002), and residual tumor lesions < 1 cm (*P* = 0.001) were associated with longer DFS, whereas only age ≤ 50 years (*P* = 0.034) and residual tumor lesions < 1 cm (*P* = 0.006) were associated with longer OS. Positive ascites cytology (*P* = 0.027) was associated with poor OS. In the multivariate Cox proportional hazards regression analysis, age > 50 years (HR, 2.61; 95% CI, 1.12–6.08; *P* = 0.026) and FIGO stage III or IV (HR, 5.48; 95% CI, 1.21–24.71; *P* = 0.027) were independent prognostic risk factors for DFS, whereas residual tumor lesions < 1 cm (HR, 0.32; 95% CI, 0.15–0.70; *P* = 0.004) was an independent prognostic protective factor for DFS. Patients with residual tumor lesions < 1 cm (HR, 0.19; 95% CI, 0.07–0.51; *P* = 0.001) had a longer OS, while those aged > 50 years (HR, 4.94; 95% CI, 1.58–15.44; *P* = 0.006) had a shorter OS. Multivariate Cox proportional hazards regression analysis showed that pelvic lymph node histological status was not associated with DFS (HR, 2.26; 95% CI, 0.64–7.94; *P* = 0.205) or OS (HR, 4.57; 95% CI, 0.70–30.02; *P* = 0.114) in the lymphadenectomy cohort. The results are presented in Tables 2 and 3.

**Table 2:** Univariable Cox proportional hazard regression analysis of DFS and OS in the post-PSM cohort.

	DFS		OS	
Characteristic	HR (95%CI)	*P* value	HR (95%CI)	*P* value
**Age, year**		0.020		0.034
≤50	Reference		Reference	
>50	2.38 (1.15–4.95)		3.06 (1.09–8.60)	
**FIGO (2014)**		0.002		0.049
I and II	Reference		Reference	
III and IV	9.68 (2.30–40.68)		4.40 (1.01–19.19)	
**CA-125, U/mL**		0.587		0.666
≤35	Reference		Reference	
>35	1.49 (0.36–6.23)		1.56 (0.21–11.72)	
**Operation method**		0.123		0.263
Laparotomy	Reference		Reference	
Laparoscopy	0.04 (0.01–2.41)		0.04 (0.02–11.40)	
**Tumor size, cm**		0.736		0.186
≤8	Reference		Reference	
>8	1.13 (0.56–2.27)		0.53 (0.20–1.36)	
Pathological consistency		0.149		0.403
Consistent	1.07 (0.40–2.87)	0.899	1.71 (0.38–7.69)	0.488
Not consistent	Reference		Reference	
Unknown	2.27 (0.76–6.76)	0.143	2.89(0.56–14.89)	0.205
**Debulking surgery**		0.001		0.006
Optimal (<1 cm)	0.28 (0.14–0.57)		0.27 (0.11–0.69)	
Suboptimal (≥1 cm)	Reference		Reference	
**Ascites cytology**		0.165		0.080
Positive	3.04 (0.96–9.62)	0.058	11.24 (1.31–96.29)	0.027
Negative	Reference		Reference	
Unknown	1.96 (0.74–5.23)	0.178	6.11 (0.79–47.03)	0.082
**Pelvic LN**		0.037		0.069
Positive	2.65 (0.84–8.37)	0.096	2.89 (0.48–17.31)	0.245
Negative	Reference		Reference	
Unknown	3.66 (1.36–9.82)	0.010	5.44 (1.22–24.19)	0.026
**Para-aortic LN**		0.094		
Positive	1.87 (0.31–11.22)	0.493	–	
Negative	Reference		–	
Unknown	3.53 (1.07–11.71)	0.039		
**Adjuvant therapy**		0.024		0.209
None	Reference		Reference	
Chemotherapy	5.19 (1.24–21.73)		0.62 (0.30–1.30)	

PSM, propensity score matching; DFS, disease-free survival; OS, overall survival; HR, hazard ratio; CI, confidence interval; FIGO, International Federation of Gynecology and Obstetrics, CA-125, cancer antigen 125; LN, lymph node.

**Table 3:** Multivariate Cox proportional hazard regression analysis for DFS and OS in the post-PSM cohort.

	DFS		OS	
Characteristic	HR (95%CI)	*P* value	HR (95%CI)	*P* value
**Age (Year)**		0.026		0.006
≤50	Reference		Reference	
>50	2.61 (1.12–6.08)		4.94 (1.58–15.44)	
**FIGO (2014)**		0.027		
I and II	Reference			
III and IV	5.48 (1.21–24.71)			
**Debulking surgery**		0.004		0.001
Optimal (<1 cm)	0.32 (0.15–0.70)		0.19 (0.07–0.51)	
Suboptimal (≥1 cm)	Reference		Reference	
**Pelvic LN**		0.017		0.049
Positive	2.26 (0.64–7.94)	0.205	4.57 (0.70–30.02)	0.114
Negative	Reference		Reference	
Unknown	4.20 (1.52–11.61)	0.006	6.59 (1.46–29.82)	0.014

PSM, propensity score matching; DFS, disease-free survival; OS, overall survival; HR, hazard ratio; CI, confidence interval; FIGO, International Federation of Gynecology and Obstetrics; LN, lymph node.

### Subgroup analysis stratified by FIGO staging

We also conducted a subgroup analysis stratified by FIGO staging. We performed PSM for patients with FIGO stage I and II disease and for those with FIGO stage III and IV disease. The basic clinical characteristics of the pre- and post-PSM cohorts are presented in Supplementary **Tables S2** and **S3**. In the pre-PSM cohort, there existed a statistically significant difference in adjuvant therapy (*P* = 0.002) in the FIGO stage I and II disease groups and statistical differences in age (*P* = 0.008) and operation method (*P* = 0.020) in the FIGO stage III and IV disease groups. The patients in each group were matched using PSM at a 1:1 ratio. A total of 24 women were included in the post-PSM cohort of FIGO stage I and II disease groups, with 12 (50%) each in the lymphadenectomy and no lymphadenectomy groups. A total of 58 women were included in the post-PSM cohort in FIGO stage III and IV disease groups, with 29 (50%) each in the lymphadenectomy and no lymphadenectomy groups. The patients’ basic characteristics were not statistically different in the post-PSM cohort (*P* > 0.05).

**Fig. 2:**
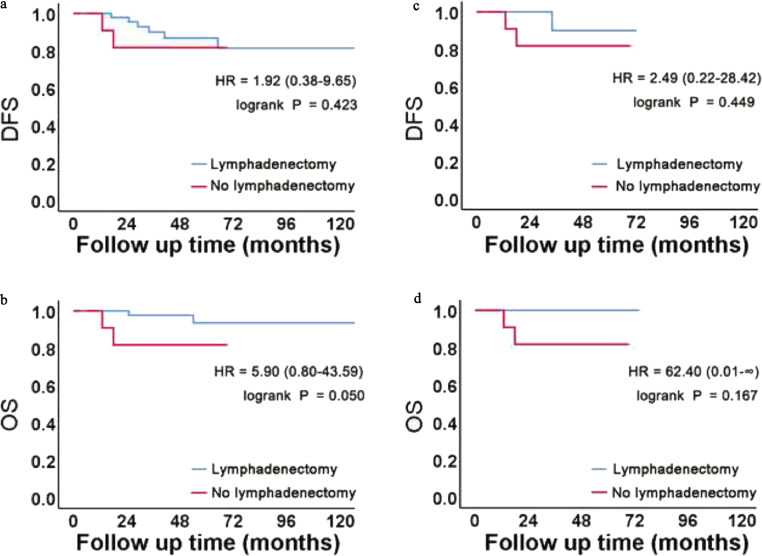
Kaplan-Meier curves of patients with FIGO stage I and II disease in the pre- and post-PSM cohorts. (A, B) In the pre-PSM cohort, Kaplan-Meier survival analysis revealed that lymphadenectomy may have a protective effect on OS. (C, D) After matching, we found no significant survival benefit from lymphadenectomy.

**Fig. 3:**
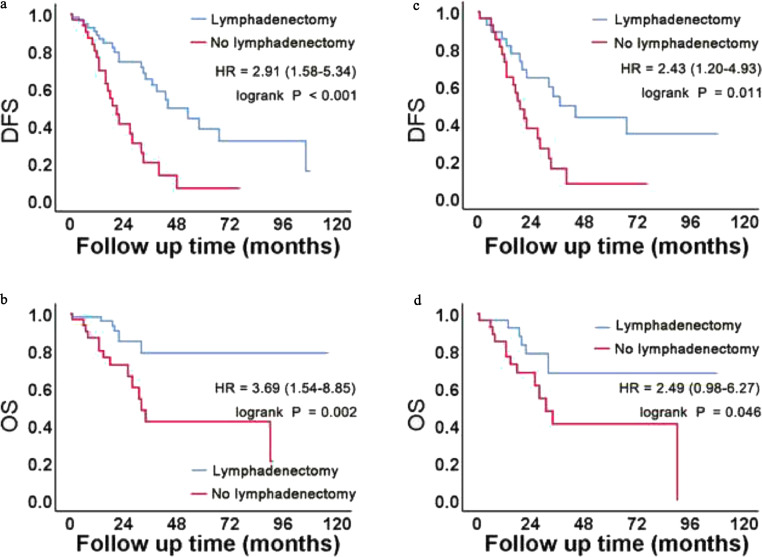
Kaplan-Meier curves of patients with FIGO stage III and IV disease in the pre- and post-PSM cohorts. (A, B) In the pre-PSM cohort, Kaplan-Meier survival analysis revealed that lymphadenectomy had a protective effect on DFS and OS. (C, D) After matching, we found statistical differences in DFS and OS between the two groups.

Kaplan-Meier survival curves for OS and DFS analyzed according to the lymphadenectomy status in each subgroup are presented in Figs. 2 and 3, respectively. In the pre-PSM cohort, Kaplan-Meier analyses showed that lymphadenectomy had a significant protective effect on DFS (*P* < 0.001) and OS (*P* = 0.002) for those in those in FIGO stage III and IV disease groups. The median DFS of the two pre-PSM cohorts was 44 and 21 months, respectively, and the median OS of the no lymphadenectomy group was 32 months. For patients in FIGO stage I and II disease groups, lymphadenectomy may have a protective effect on OS (*P* = 0.050), but no significant difference was noted for DFS (*P* = 0.423). These results are shown in Figs. 2**A**, 2**B**, 3**A**, and 3**B**. In the post-PSM cohort, no significant differences in DFS (*P* = 0.449) or OS (*P* = 0.167) were found based on the lymphadenectomy status for those in the FIGO stage I and II disease groups. In the FIGO stage III and IV disease groups, DFS (*P* = 0.011) and OS (*P* = 0.046) significantly improved in the lymphadenectomy group. The median DFS of the two post-PSM cohorts was 37 and 19 months, respectively, and the median OS of the no lymphadenectomy group was 31 months. These results are shown in Figs. 2**C**, 2**D**, 3**C**, and 3**D**.

## Discussion

### Main findings

We performed a multicenter retrospective study to evaluate the effects of lymphadenectomy on the prognosis of patients with LGSOC. After rigorous matching of clinical characteristics, we found that the patients benefited significantly from lymphadenectomy. To assess the impact of FIGO stage on the prognosis of systematic lymphadenectomy, a subgroup analysis stratified by FIGO staging was performed. Patients with early and advanced LGSOC were divided into groups based on whether they underwent systematic lymphadenectomy or not. The results showed that there was no significant difference in survival outcomes between the two groups in the early LGSOC cohort, but there were significant differences in survival outcomes between the two groups in the advanced LGSOC cohort. The prognosis of patients with LGSOC was mainly related to age, FIGO stage, and optimal cytoreductive surgery.

### Strengths and limitations

In our study, the median DFS and OS were consistent with those in previous studies [2,14,16]. Among women who underwent lymphadenectomy, 27.3% (30/110) had lymph node metastases. This study also had some limitations. Due to the rarity of the disease, data volumes were insufficient, even for patients enrolled in multiple medical centers. Most of the patients with early LGSOC underwent lymphadenectomy which may have affected the results of the subgroup analysis, and some statistical results could not be calculated owing to the limitation of data quantity. The main limitation of this study was its retrospective nature. Additionally, some patients with advanced ovarian cancer died after surgery due to intestinal obstruction or other complications, and some patients were lost to follow-up bacause the study covered a 10-year period. We also did not discuss the preoperative lymph node status indicated by radiological examination and intraoperative exploration, which may represent selection bias. We did not know how gynecologists make surgical decisions, and the influence of the surgical scope of lymphadenectomy on prognosis was not analyzed. Finally, we did not discuss the postoperative complications associated with lymphadenectomy.

However, the strength of our study lies in its large sample of patients from four medical centers, which improved the authenticity and reliability of the data analysis. Such large amounts of data are rare for rare diseases. All patients were diagnosed and surgically treated by gynecologists, and the pathological diagnosis was evaluated and confirmed by senior pathology physicians. To further increase the credibility of the results, we matched the basic clinical characteristics using PSM to fit prospective clinical trials and explore the impact of lymphadenectomy on prognosis. We also performed subgroup analysis to explore the clinical benefits of lymphadenectomy based on the FIGO stage.

### Interpretation

Compared to HGSOC, LGSOC is relatively rare in clinical practice, and appropriate corresponding diagnosis and treatment strategies lack strong evidence. LGSOC is characterized by a slow growth pattern and insensitivity to chemotherapy [4]. Therefore, initial cytoreductive surgery is more significant for LGSOC than for HGSOC [26]. Data from Gynecologic Oncology Group (GOG) 182 study on 189 patients with LGSOC showed that patients with residual lesions > 1 cm after initial cytoreductive surgery had significantly shorter DFS (14.1 months vs 33.2 months, *P* < 0.001) and OS (42.0 months vs 96.9 months, *P* < 0.001) compared to those with microscopic residual lesions [27]. Most scholars would agree that it is ideal to remove as many macroscopic tumor lesions as possible, if not all, during initial cytoreductive surgery for LGSOC [4,11,12,15]. Similarly, in our study, optimal cytoreductive surgery was closely associated with longer DFS (*P* = 0.004) and OS (*P* = 0.001). However, whether performing lymphadenectomy along with cytoreductive surgery for the initial surgical treatment improves the survival outcomes of patients with LGSOC remains inconclusive.

Our study found that, for patients with LGSOC, lymphadenectomy had survival benefits, which is consistent with the findings of some previous studies. In related studies on ovarian cancer, lymphadenectomy was not found to provide significant benefits to patients. A prospective randomized trial of patients with advanced ovarian cancer showed that the removal of enlarged lymph nodes and systematic lymphadenectomy resulted in significantly longer DFS but had no statistical effect on OS [23]. Our study did not discuss whether enlarged lymph nodes were explored before or during surgery, which may have resulted in selection bias. A randomized study of systematic lymphadenectomy and sampling in early ovarian cancer showed that systematic lymphadenectomy contributed to staging with no survival benefit [24]. A randomized trial of lymphadenectomy in 647 patients with advanced epithelial ovarian cancer (the LION study) showed that if there were no obviously enlarged lymph nodes before and during surgery, lymphadenectomy did not offer any survival benefits [22]. These studies were prospective randomized clinical trials with high authenticity and reliability; however, most of the patients had HGSOC, and only a few had LGSOC.

Gockley et al. [28]. used the National Cancer Database to analyze 404 patients who were matched by lymphadenectomy and showed that not performing lymphadenectomy was associated with an increased risk of death. The authors also used PSM to balance differences in patients’ basic characteristics; however, due to data limitations, no disease recurrence-related assessment was conducted. Simon et al. [17]. retrospectively analyzed the effect of lymphadenectomy on PFS and OS in 126 patients with LGSOC and found no significant improvement in prognosis, with similar results in the subgroup analysis. The results are presented in Supplementary **Table S4**. Based on previous research, we included 155 patients from four centers, used PSM to balance the clinical characteristics of the patients, and performed a subgroup analysis stratified by FIGO staging. Therefore, our statistical analysis was both highly rigorous and accurate in evaluating the role of lymphadenectomy in the prognosis of patients with LGSOC.

Ovarian cancer significantly affects the survival of women. In patients with LGSOC, it is ideal to remove all macroscopic tumor lesions; however, whether systematic lymphadenectomy significantly affects survival remains controversial. Subgroup analysis of early LGSOC in this study showed that systematic lymphadenectomy did not improve DFS (*P* = 0.449) or OS (*P* = 0.167), and there was no statistical difference in survival prognosis between the lymphadenectomy and no lymphadenectomy groups. For patients with early clinical stage, systematic lymphadenectomy may not be performed after systematic preoperative imaging examination and intraoperative evaluation; however, occult lymph node metastasis should still be vigilant.

Subgroup analysis of advanced LGSOC showed that systematic lymphadenectomy in patients with advanced LGSOC improved DFS (*P* = 0.011) and OS (*P* = 0.046), and the prognosis of the two groups was statistically different, which was consistent with the results of previous studies. Among patients with advanced LGSOC, 68.5% (37/54) of those in the optimal cytoreductive surgery group (maximum residual disease < 1 cm) underwent systematic lymphadenectomy, while 56.8% (21/37) of those in the suboptimal cytoreductive surgery group (maximum residual lesion > 1 cm) underwent systematic lymphadenectomy. Multivariate Cox proportional hazards regression analysis showed that the histological status of pelvic lymph nodes was not associated with DFS (*P* = 0.205) or OS (*P* = 0.114) in the lymphadenectomy cohort. Considering the insensitivity of the disease to chemotherapy, there was a higher rate of lymph node metastases in advanced patients, and systematic lymphadenectomy was associated with tumor reduction, thereby improving patient outcomes. However, in patients with a large postoperative residual disease, the beneficial impact of systematic lymphadenectomy is questionable.

Therefore, we believe that systematic lymphadenectomy can improve the DFS and OS of patients with advanced LGSOC. These results may be useful for surgical decision-making in clinical settings. Patients with early clinical presentation may refuse lymphadenectomy to avoid postoperative complications. Although prospective multicenter studies are needed to confirm these findings, we recognize that this may be difficult because LGSOC is rare.

## Conclusion

LGSOC is a rare malignant ovarian tumor. Despite considerable efforts over the past few decades, there is still a lack of precise guidance on surgical diagnosis and treatment. Our study suggests that for LGSOC patients in the early clinical stage, systemic lymphadenectomy can be avoided after adequate preoperative and intraoperative evaluation; however, occult lymph node metastasis still needs to be vigilant. Systematic lymphadenectomy can improve DFS and OS in patients with advanced LGSOC. However, in patients with large postoperative residual disease, the beneficial impact of systematic lymphadenectomy warrants further investigation. These results may influence surgical decision-making regarding treatment options for LGSOC. We recommend that all patients with LGSOC undergo a detailed preoperative evaluation to accurately formulate a surgical treatment plan thus improving patients’ prognosis.

## Funding

This work was supported by the National Key Technology R&D Program of China (grant numbers 2019YFC1005200 and 2019YFC1005204), the Taishan Scholar Youth Project of Shandong Province (grant number tsqn201812130), and the Research Leader Studio of Jinan (grant number 2019GXRC049).

## Ethics approval and consent to participate

This retrospective study was approved by the Ethical Committee of Qilu Hospital of Shandong University (protocol number KYLL-202011-158-1) and obtained a waiver for informed consent. Before the analysis, the privacy of each patient was maintained.

## Availability of data and materials

The datasets used and/or analysed during the current study are available from the corresponding author on reasonable request.

## CRediT authorship contribution statement

**Zhongshao Chen:** Conceptualization, Methodology, Investigation, Formal analysis, Writing – original draft, Writing – review & editing, Visualization. **Ran Chu:** Conceptualization, Methodology, Formal analysis, Writing – review & editing. **Yuanming Shen:** Conceptualization, Investigation, Resources, Data curation. **Qin Yao:** Conceptualization, Investigation, Resources, Data curation. **Jingying Chen:** Conceptualization, Methodology, Formal analysis, Writing – review & editing. **Tianyu Qin:** Investigation, Resources. **Li Li:** Conceptualization, Methodology, Writing – review & editing. **Gang Chen:** Conceptualization, Investigation, Resources, Data curation. **Qinglei Gao:** Resources, Data curation. **Chaoyang Sun:** Conceptualization, Investigation, Resources, Data curation. **Li Song:** Conceptualization, Resources. **Junting Li:** Investigation, Resources. **Penglin Liu:** Investigation, Resources. **Xiyu Pan:** Conceptualization, Investigation, Resources. **Jingnan Li:** Investigation, Resources. **Xiaoying Zhu:** Investigation, Resources. **Li Zhang:** Investigation, Resources. **Xu Qiao:** Methodology, Formal analysis. **Ding Ma:** Conceptualization. **Beihua Kong:** Conceptualization, Methodology. **Kun Song:** Conceptualization, Investigation, Resources, Data curation, Methodology, Writing – review & editing, Supervision.

## Declaration of Competing Interest

All authors declare no conflict of interest.

## Appendix Supplementary materials

Image, application 1
