# Supplementary material for: Evaluation of the prognostic value of lymphadenectomy for low-grade serous ovarian cancer: A case-control multicenter retrospective study
Source: Transl Oncol. 2022 Jul 4;23:101476. doi: 10.1016/j.tranon.2022.101476 (PMC9263964; doi:10.1016/j.tranon.2022.101476)
Supplement: Supplementary file 1 [file mmc1.docx]

**Supplemental Material**

The supplemental material includes:

- **Fig S1**
- **Table S1 - Table S4**

**Fig S1: The workflow of this study.**


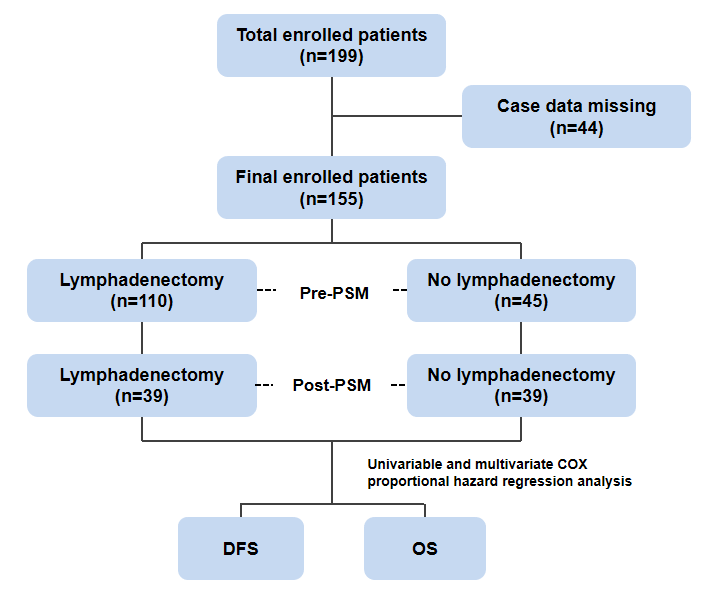


DFS, disease-free survival; OS, overall survival

Table S1: Histological status of lymph nodes in patients with lymphadenectomy.

| **Characteristic** | **Patients**  n=110 (71.0%)  n (%) |
| --- | --- |
| **Histological status of LN** |  |
| N+ | 30 (27.3%) |
| N- | 80 (72.7%) |
| **Histological status of pelvic LN** |  |
| N+ | 24 (22.0%) |
| N- | 85 (78.0%） |
| **Histological status of para-aortic LN** |  |
| N+ | 17 (23.3%) |
| N- | 56 (76.7%） |

LN, lymph node.

Table S2. Characteristics of patients in the pre- and post-PSM cohorts (FIGO stage I and II).

|  | **Before Matching (n=64)** | | |  | **After Matching (n=24)** | | |
| --- | --- | --- | --- | --- | --- | --- | --- |
| **Characteristic** | **Lymphadenectomy**  **(n=52)** | **No lymphadenectomy**  **(n=12)** | ***P* value** |  | **Lymphadenectomy**  **(n=12)** | **No lymphadenectomy**  **(n=12)** | ***P* value** |
| **Age, year** |  |  | 1.000 |  |  |  | 1.000 |
| ≤50 | 34 (65.4) | 8 (66.7) |  |  | 8 (66.7) | 8 (66.7) |  |
| >50 | 18 (34.6) | 4 (33.3) |  |  | 4 (33.3) | 4 (33.3) |  |
| **FIGO (2014)** |  |  | 1.000 |  |  |  | 1.000 |
| I | 42 (80.8) | 10 (83.3) |  |  | 10 (83.3) | 10 (83.3) |  |
| II | 10 (19.2) | 2 (16.7) |  |  | 2 (16.7) | 2 (16.7) |  |
| **CA-125, U/mL** |  |  | 0.077 |  |  |  | 0.155 |
| ≤35 | 21 (40.4) | 1 (8.3) |  |  | 5 (41.7) | 1 (8.3) |  |
| >35 | 31 (59.6) | 11 (91.7) |  |  | 7 (58.3) | 11 (91.7) |  |
| **Operation method** |  |  | 0.130 |  |  |  | 0.408 |
| Laparotomy | 40 (76.9) | 6 (50.0) |  |  | 8 (75.0) | 6 (50.0) |  |
| Laparoscopy | 12 (23.1) | 6 (50.0) |  |  | 4 (25.0) | 6 (50.0) |  |
| **Tumor size, cm** |  |  | 0.588 |  |  |  | 0.667 |
| ≤8 | 32 (61.5) | 9 (75.0) |  |  | 7 (58.3) | 9 (75.0) |  |
| >8 | 20 (38.5) | 3 (25.0) |  |  | 5 (41.7) | 3 (25.0) |  |
| **Pathological consistency** |  |  | 0.459 |  |  |  | 1.000 |
| Consistent | 37 (71.2) | 7 (58.3) |  |  | 7 (58.3) | 7 (58.3) |  |
| Not consistent | 9 (17.3) | 4 (33.3) |  |  | 4 (33.3) | 4 (33.3) |  |
| Unknown | 6 (11.5) | 1 (8.3) |  |  | 1 (8.3) | 1 (8.3) |  |
| **Debulking surgery** |  |  | 0.574 |  |  |  | 1.000 |
| Optimal (<1cm) | 49 (94.2) | 11 (91.7) |  |  | 12 (100.0) | 11 (91.7) |  |
| Suboptimal (≥1cm) | 3 (5.8) | 1 (8.3) |  |  | 0 (0.0) | 1 (8.3) |  |
| **Ascites cytology** |  |  | 0.879 |  |  |  | 0.662 |
| Positive | 9 (17.3) | 2 (16.7) |  |  | 1 (8.3) | 2 (16.7) |  |
| Negative | 22 (42.3) | 6 (50.0) |  |  | 5 (41.7) | 6 (50.0) |  |
| Unknown | 21 (40.4) | 4 (33.3) |  |  | 6 (50.0) | 4 (33.3) |  |
| **Adjuvant therapy** |  |  | 0.002 |  |  |  | 1.000 |
| None | 9 (18.8) | 8 (66.7) |  |  | 8 (66.7) | 8 (66.7) |  |
| Chemotherapy | 43 (81.3) | 4 (33.3) |  |  | 4 (33.3) | 4 (33.3) |  |

Values are presented as n (%).

PSM, propensity score matching; FIGO, International Federation of Gynecology and Obstetrics; CA-125, carbohydrate antigen 125

Table S3. Characteristics of patients in the pre- and post-PSM cohorts (FIGO stage III and IV).

|  | **Before Matching (n=91)** | | |  | **After Matching (n=58)** | | |
| --- | --- | --- | --- | --- | --- | --- | --- |
| **Characteristic** | **Lymphadenectomy**  **(n=58)** | **No lymphadenectomy**  **(n=33)** | ***P* value** |  | **Lymphadenectomy**  **(n=29)** | **No lymphadenectomy**  **(n=29)** | ***P* value** |
| **Age, year** |  |  | 0.008 |  |  |  | 1.000 |
| ≤50 | 41 (70.7) | 14 (42.4) |  |  | 14 (48.3) | 14 (48.3) |  |
| >50 | 17 (29.3) | 19 (57.6) |  |  | 15 (51.7) | 15 (51.7) |  |
| **FIGO (2014)** |  |  | 1.000 |  |  |  | 1.000 |
| I | 53 (91.4) | 30 (90.9) |  |  | 26 (89.7) | 26 (89.7) |  |
| II | 5 (8.6) | 3 (9.1) |  |  | 3 (10.3) | 3 (10.3) |  |
| **CA-125, U/mL** |  |  | 1.000 |  |  |  | 1.000 |
| ≤35 | 5 (8.6) | 2 (6.1) |  |  | 2 (6.9) | 2 (6.9) |  |
| >35 | 53 (91.4) | 31 (93.9) |  |  | 27 (93.1) | 27 (93.1) |  |
| **Operation method** |  |  | 0.020 |  |  |  | 1.000 |
| Laparotomy | 47 (81.0) | 33 (100.0) |  |  | 29 (100.0) | 29 (100.0) |  |
| Laparoscopy | 11 (19.0) | 0 (0.0) |  |  | 0 (0.0) | 0 (0.0) |  |
| **Tumor size, cm** |  |  | 0.824 |  |  |  | 0.100 |
| ≤8 | 26 (44.8) | 14 (42.4) |  |  | 7 (24.1) | 14 (48.3) |  |
| >8 | 32 (55.2) | 19 (57.6) |  |  | 22 (75.9) | 15 (51.7) |  |
| **Pathological consistency** |  |  | 0.582 |  |  |  | 0.949 |
| Consistent | 35 (60.3) | 20 (60.6) |  |  | 19 (65.5) | 18 (62.1) |  |
| Not consistent | 13 (22.4) | 5 (15.2) |  |  | 4 (13.8) | 4 (13.8) |  |
| Unknown | 10 (17.2) | 8 (24.2) |  |  | 6 (20.7) | 7 (24.1) |  |
| **Debulking surgery** |  |  | 0.252 |  |  |  | 0.279 |
| Optimal (<1cm) | 37 (63.8) | 17 (51.5) |  |  | 20 (69.0) | 16 (55.2) |  |
| Suboptimal (≥1cm) | 21 (36.2) | 16 (48.5) |  |  | 9 (31.0) | 13 (44.8) |  |
| **Ascites cytology** |  |  | 0.097 |  |  |  | 0.269 |
| Positive | 7 (12.1) | 9 (27.3) |  |  | 3 (10.3) | 7 (24.1) |  |
| Negative | 15 (25.9) | 4 (12.1) |  |  | 6 (20.7) | 3 (10.3) |  |
| Unknown | 36 (62.1) | 20 (60.6) |  |  | 20 (69.0) | 19 (65.5) |  |
| **Adjuvant therapy** |  |  | 0.619 |  |  |  | 1.000 |
| None | 2 (3.4) | 2 (6.1) |  |  | 2 (6.9) | 1 (3.4) |  |
| Chemotherapy | 56 (96.6) | 31 (93.9) |  |  | 27 (93.1) | 28 (96.6) |  |

Values are presented as n (%).

PSM, propensity score matching; FIGO, International Federation of Gynecology and Obstetrics; CA-125, carbohydrate antigen

**Table S4. Studies concerning prognosis of lymph node dissection in patients with OC/LGSOC.**

| **Author** | **Simon V, 2020^17^** | | **Gockley A, 2017^28^** | | **Harter P, 2019^22^** | | **Panici PB, 2005^23^** | | **Maggioni A, 2006^24^** | | **Our research (Before PSM)** | | **Our research (After PSM)** | |
| --- | --- | --- | --- | --- | --- | --- | --- | --- | --- | --- | --- | --- | --- | --- |
| **Grouping** | LND- | LND+ | LND- | LND+ | LND- | LND+ | bulky nodes+ | LND+ | LN sampling | LND+ | LND- | LND+ | LND- | LND+ |
| **Year** | 58 | 51 | 55.54 | 54.08 | 60 | 60 | 56 | 53 | 52 | 51- | 49.7 | 46.4 | 47.6 | 51.2 |
| **No.patients** | 31 | 91 | 202 | 202 | 324 | 323 | 211 | 216 | 130 | 138 | 45 | 110 | 39 | 39 |
| **Histological types** | LGSOC | LGSOC | LGSOC | LGSOC | AOC | AOC | AOC | AOC | OC | OC | LGSOC | LGSOC | LGSOC | LGSOC |
| **FIGO criteria** | FIGO | FIGO | FIGO 2014 | FIGO 2014 | FIGO | FIGO | FIGO | FIGO | FIGO | FIGO | FIGO 2014 | FIGO 2014 | FIGO 2014 | FIGO 2014 |
| **FIGO stage** |  |  |  |  |  |  |  |  |  |  |  |  |  |  |
| **I** | 6  (20.7) | 11  (12.2) | - | - | 17  (5.2) | 15  (4.6) | - | - | 90  (69.2) | 102  (73.9) | 10  (22.2) | 42  (38.2) | 10  (25.6) | 9  (23.1) |
| **II** |  |  | - | - | 52  (16.0) | 41  (12.7) | - | - | 39  (30.0) | 33  (23.9) | 2  (4.4) | 10  (9.1) | 2  (5.1) | 2  (5.1) |
| **III** | 23  (79.3) | 79  (87.8) | 171  (84.7) | 165  (81.7) | 24  (75.3) | 261  (80.8) | 199  (94.3) | 207  (95.8) | - | - | 30  (66.7) | 53  (48.2) | 25  (64.1) | 26  (66.7) |
| **IV** |  |  | 31  (15.3) | 37  (18.3) | 11  (3.4) | 6  (1.9) | 12  (5.7) | 9  (4.2) | - | - | 3  (6.7) | 5  (4.5) | 2  (5.1) | 2  (5.1) |
| **LN status** |  |  |  |  |  |  |  |  |  |  |  |  |  |  |
| **pN+** | - | 58.2% | - | - | - | 55.7% | 42% | 70% | 5% | 15% | - | 27.3% | - | 53.8% |
| **pN-** | - | 41.8% | - | - | - | 44.3% | 58% | 30% | 95% | 85% | - | 72.7% | - | 46.2% |
| **Follow-up**  **(months)** | 27.5 | | 72.7 | | 72 | | 68.4 | | 87.8 | | 32 | 40 | 30 | 44 |
| **Median DFS/PFS (months)** | 41 | | - | - | 25.5 | 25.5 | 22.4 | 29.4 | - | - | 27 | 106 | 27 | - |
| **5-year DFS /PFS** | 41% (31.2–54.1%) | | - | - | - | - | 21.6% | 31.2% | 71.3% | 78.3% | 26.3% | 64.5% | 35.0% | 62.2% |
| **Median OS (months)** | 130 | | 58 | 106.5 | 69.2 | 65.5 | 56.3 | 58.7 | - | - | 90 | - | - | - |
| **5-year OS** | 77% (68.3–87.1%) | | - | - | - | - | 47% | 48.5% | 81.3% | 84.2% | 54.5% | 86.3% | 56.2% | 82.9% |

Values are presented as n(%) or median (range).

PSM, propensity score matching; LND, lymph node dissection; LN, lymph node; LGSOC, low grade serous ovarian cancer; AOC, advanced ovarian cancer; OC, ovarian cancer; FIGO, International Federation of Gynecology and Obstetrics; pN, pathological lymph node status; PFS, progression-free survival; DFS, disease-free survival; OS, Overall survival.
